# Serum butyrylcholinesterase predicts survival after extracorporeal membrane oxygenation after cardiovascular surgery

**DOI:** 10.1186/cc13711

**Published:** 2014-01-30

**Authors:** Klaus Distelmaier, Max-Paul Winter, Kurt Rützler, Gottfried Heinz, Irene M Lang, Gerald Maurer, Herbert Koinig, Barbara Steinlechner, Alexander Niessner, Georg Goliasch

**Affiliations:** 1 Department of Internal Medicine II, Medical University of Vienna, Waehringer Guertel 18-20, A-1090 Vienna, Austria; 2 Division of Endocrinology, Mayo Clinic College of Medicine, Rochester, USA; 3 Department of Cardiothoracic and Vascular Anaesthesia and Intensive Care Medicine, Medical University of Vienna, Vienna, Austria; 4 Department of Anesthesia and Intensive Care Medicine, University Hospital Zürich, Zürich, Switzerland; 5 Department of Anesthesia and Intensive Care Medicine, Landesklinikum Krems, Krems, Austria; 6 Zena and Michael A. Wiener Cardiovascular Institute, Mount Sinai School of Medicine, New York, NY, USA

## Abstract

**Introduction:**

Risk stratification in patients undergoing extracorporeal membrane oxygenation (ECMO) support after cardiovascular surgery remains challenging, because data on specific outcome predictors are limited. Serum butyrylcholinesterase demonstrated a strong inverse association with all-cause and cardiovascular mortality in non-critically ill patients. We therefore evaluated the predictive value of preoperative serum butyrylcholinesterase levels in patients undergoing venoarterial ECMO support after cardiovascular surgery.

**Methods:**

We prospectively included 191 patients undergoing venoarterial ECMO therapy after cardiovascular surgery at a university-affiliated tertiary care center in our registry.

**Results:**

All-cause and cardiovascular mortality were defined as primary study end points. During a median follow-up time of 51 months (IQR, 34 to 71) corresponding to 4,197 overall months of follow-up, 65% of patients died. Cox proportional hazard regression analysis revealed a significant and independent inverse association between higher butyrylcholinesterase levels and all-cause mortality with an adjusted hazard ratio (HR) of 0.44 (95% CI, 0.25 to 0.78; *P* = 0.005), as well as cardiovascular mortality, with an adjusted HR of 0.38 (95% CI, 0.21 to 0.70; *P* = 0.002), comparing the third with the first tertile. Survival rates were higher in patients within the third tertile of butyrylcholinesterase compared with patients within the first tertile at 30 days (68% versus 44%) as well as at 6 years (47% versus 21%).

**Conclusions:**

The current study revealed serum butyrylcholinesterase as a strong and independent inverse predictor of all-cause and cardiovascular mortality in patients undergoing venoarterial ECMO therapy after cardiovascular surgery. These findings advance the limited knowledge on risk stratification in patients undergoing ECMO support and represent a valuable addition for a comprehensive decision making before ECMO implantation.

## Introduction

Extracorporeal membrane oxygenation (ECMO) is an established therapeutic option for circulatory assistance in patients with cardiogenic shock refractory to conventional medical therapies after cardiovascular surgery [1]. However, long-term prognosis in this patient population remains poor, and risk stratification, challenging, because data on specific outcome predictors are limited [2].

Serum butyrylcholinesterase previously demonstrated a strong inverse association with all-cause and cardiovascular mortality in a community-based population, as well as in patients with coronary artery disease [3,4]. Although butyrylcholinesterase was discovered more than 80 years ago [5], the precise physiological function is still widely unknown. Because butyrylcholinesterase catalyzes the hydrolysis of choline esters, including several muscle relaxants [6], the previous scientific interest has been reduced to its impact on neuromuscular blockade after the use of muscle relaxants in surgical patients over recent years [7-9].

However, based on its remarkable predictive value for mortality in noncritically ill patient populations [3,4], we propose that butyrylcholinesterase might be a powerful predictor for clinical outcome in patients undergoing ECMO therapy. Nevertheless, to the best of our knowledge, no such data exist from previous investigations. We therefore analyzed the impact of preoperative serum butyrylcholinesterase levels on cardiovascular and all-cause mortality in patients undergoing venoarterial ECMO support after cardiovascular surgery.

## Materials and methods

### Study population

We prospectively enrolled all patients undergoing venoarterial ECMO support after cardiovascular surgery between February 2002 and December 2009 at the Vienna General Hospital, a university-affiliated tertiary care center in our registry, as previously published [10]. No exclusion criteria were applied. The study protocol was reviewed and approved by the Ethics Committee of the Medical University of Vienna and conforms to the Declaration of Helsinki; all patients admitted to the Medical University of Vienna provided written consent at hospital admission. The ECMO circuit consisted of a centrifugal pump console (Bio-Console560; Medtronic, New York, NY, USA) with a hollow-fiber membrane oxygenator (Affinity-NTTM; Medtronic). An experienced perfusionist and the on-shift intensive care physician routinely serviced the ECMO circuit once daily. The circuit was changed if blood oxygenation declined sharply or if significant fibrin deposition or clots were present. Under ECMO therapy, lungs were ventilated at peak airway pressures <25 cmH_2_O, physiologic respiratory tidal volumes (6 to 8 ml/kg), a respiratory rate (≤10/min), and the fractional inspired oxygen (FiO_2_) was reduced to 0.3. FiO_2_ was adjusted by using the ECMO circuit to maintain a PO_2_ between 80 and 100 mm Hg. A more-detailed description regarding ECMO management was previously published [10].

### Clinical definitions and study end points

Cardiovascular risk factors were recorded according to the respective guidelines. Determination of serum butyrylcholinesterase activity is part of a routine laboratory test, performed in all patients at time of admission at the Vienna General Hospital. Serum butyrylcholinesterase was measured according to local laboratory standard procedures by using an enzyme-kinetic assay with butyrylthiocholine-iodide as substrate in serum at 25°C [11]. The simplified acute physiology score (SAPS) II score [12] was recorded at the time of ICU admission, and the European System of Cardiac Operative Risk Evaluation (EuroSCORE) was computed as previously described [13]. All-cause and cardiovascular mortality were selected as primary study end points and obtained by screening the national register of death, including screening for the cause of death (according to the *International Classification of Diseases,* 10^th^ Revision).

### Statistical analysis

Discrete data were presented as count and percentage and analyzed by using a χ^2^ test. Continuous data were presented as median and interquartile range (IQR) and compared by using analysis of variance (ANOVA). Their association with butyrylcholinesterase was assessed by using Spearman-Rho correlation coefficient. Cox proportional hazard regression analysis was applied to assess the effect of butyrylcholinesterase on survival. Therefore, butyrylcholinesterase was stratified into tertiles, and the results are presented as the hazard ratio (HR) for comparison of the 3^rd^ tertile with the 1^st^ tertile. To account for potential confounding effects, we adjusted for established risk factors including age, sex, SAPS II, left ventricular function, ECMO duration, hypertension, diabetes, hypercholesterolemia, estimated glomerular filtration rate (eGFR), and albumin. GFR was calculated by using the Cockcroft-Gault formula. Collinearity within the multivariate model was tested by using the variance inflation factor and indicated no significant collinearity.

The impact of butyrylcholinesterase on survival was further analyzed by using confounder-adjusted survival curves, adjusted for all aforementioned variables in the confounder model. Kaplan-Meier analysis (log-rank test) was applied to verify the time-dependent discriminative power of butyrylcholinesterase, and results were presented after 1 month, 1 year, and 6 years (reflecting the 75% percentile of the follow-up period). Continuous variables were log transformed before entering analysis. Two-sided *P* values <0.05 were used to indicate statistical significance. The STATA11 software package and SPSS 17.0 were used for all analyses.

## Results

We enrolled 191 ECMO patients with a median age of 62 years (IQR, 51 to 69 years) undergoing ECMO after cardiovascular surgery into our study. ECMO support was initiated in 56 patients after valve surgery, in 26 after coronary artery bypass graft (CABG) surgery, in 38 after combined CABG-valve surgery, in 39 patients after cardiac transplantation, and in 32 after other cardiovascular surgeries.

ECMO implantation was performed femoral-femoral in 82% of patients, subclavian-femoral in 10% of patients, and central-femoral in 8% of patients. In 14 patients (7%), ECMO implantation was performed under cardiac massage. Indications for ECMO support were weaning failure from cardiopulmonary bypass (58%), postoperative cardiogenic shock (25%), immediate posttransplant cardiac graft failure (6%), postoperative respiratory failure (2%), postoperative bleeding/tamponade with cardiogenic shock (7%), and miscellaneous conditions (3%). ECMO-related- complications occurred in 25% of patients. In detail, major bleeding was observed in 22 patients (12%), defined as cannulation-site bleeding requiring transfusion or surgical intervention, significant thrombus formation within the ECMO circuit in 13 patients (7%), and leg ischemia including compartment syndrome in 12 patients (6%). Detailed baseline characteristics are displayed in Table 1. Correlations for continuous variables and their association with cholinesterase, assessed by using Spearman–Rho correlation coefficient, are presented in Table 2.

**Table 1:** **Baseline characteristics of total ECMO study population (*n*** **= 191) and for tertiles of butyrylcholinesterase**

	**Total study population (*n*** **= 191)**	**1**^**st **^**tertile**	**2**^**nd **^**tertile**	**3**^**rd **^**tertile**	***P *** value*
Tertiles of cholinesterase, kU/l (IQR)	5.04 (3.38-6.31)	2.81 (2.35-3.39)	5.04 (4.60-5.04)	7.45 (6.57-8.40)	**------**
Age, median years (IQR)	62 (51–69)	65 (52–70)	61 (50–70)	62 (52–69)	0.697
Male sex *n* (%)	136 (71%)	48 (75%)	44 (68%)	44 (71%)	0.613
SAPS II at ICU admission, *n* (%)	44 (31–60)	47 (37–57)	47 (33–58)	47 (31–61)	0.790
EuroSCORE (additive), points (IQR)	10 (6–13)	12 (8–13)	10 (6–14)	8 (5–11)	**0.017**
Procedure duration, hour (IQR)	7.2 (4.8-9.0)	7.0 (4.7- 8.5)	7.1 (5.3-9.0)	7.8 (4.8-9.5)	0.265
ECMO duration, median days (IQR)	4 (2–6)	6 (3–7)	5 (2–7)	4 (2–7)	0.279
Time on mechanical ventilation, median days (IQR)	11 (4–19)	9 (3–16)	13 (6–24)	10 (4–19)	0.427
IABP, *n* (%)	29 (15%)	9 (14%)	11 (17%)	9 (15%)	0.940
Hypertension*, n* (%)	86 (45%)	24 (38%)	24 (37%)	38 (61%)	**0.008**
Diabetes, *n* (%)	42 (22%)	12 (19%)	17 (26%)	13 (21%)	0.757
Hypercholesterolemia, *n* (%)	54 (28%)	11 (17%)	22 (34%)	21 (34%)	**0.037**
Coronary artery disease, *n* (%)	75 (42%)	25 (41%)	26 (43%)	24 (41%)	0.963
Left ventricular ejection fraction					
30% to 44%, *n* (%)	32 (17%)	13 (20%)	10 (15%)	9 (15%)	0.383
<30%, *n* (%)	81 (42%)	28 (44%)	31 (48%)	22 (36%)	0.354
Creatinine, mg/dl (IQR)	1.36 (1.10–1.88)	1.56 (1.17-2.23)	1.47 (1.11-1.94)	1.25 (1.03-1.69)	0.245
Estimated GFR, ml/min/1.73 m^2^ (IQR)	57 (38–77)	53 (35–74)	58 (37–67)	64 (45–88)	**0.045**
Albumin, g/L (IQR)	36 (28–42)	28 (23–35)	37 (31–41)	42 (38–45)	**<0.001**
ASAT, U/L (IQR)	39 (26–111)	48 (32–138)	48 (28–123)	31 (24–48)	0.345
ALAT, U/L (IQR)	32 (18—61)	34 (15–81)	36 (20–58)	29 (18–49)	0.486
Gamma-GT, U/L (IQR)	59 (33—121)	76 (28–151)	75 (39–149)	47 (31–88)	0.089

Continuous data are presented as median (interquartile range). *Tertiles of butyrylcholinesterase are compared by using ANOVA analysis. Categoric data are analyzed by using a test for linear association (Mantel–Haenszel χ^2^ test). ECMO, extracorporeal membrane oxygenation; GFR, glomerular filtration rate estimated by using Cockcroft-Gault formula; IABP, intraaortic balloon pump; SAPS II, simplified acute physiology score II.

Bold typefaces indicate statistical significance (*P* < 0.05).

**Table 2:** Correlation table for continuous variables and their association with cholinesterase assessed by using Spearman–Rho correlation coefficient

	**Total study population (*n*** **= 191)**	**Correlation, *r***	**P value**
Age, median years (IQR)	62 (51–69)	-0.05	0.535
SAPS II at ICU admission, *n* (%)	44 (31–60)	-0.05	0.530
EuroSCORE (additive), points (IQR)	10 (6–13)	-0.23	**0.001**
Procedure duration, hours (IQR)	7.2 (4.8-9.0)	0.12	0.113
ECMO duration, median days (IQR)	4 (2–6)	-0.17	**0.016**
Time on mechanical ventilation, median days (IQR)	11 (4–19)	0.06	0.419
Creatinine, mg/dl (IQR)	1.36 (1.10–1.88)	-0.12	0.098
Estimated GFR, ml/min/1.73 m^2^ (IQR)	57 (38–77)	0.11	0.138
Albumin, g/L (IQR)	36 (28–42)	0.59	**<0.001**
ASAT, U/L (IQR)	39 (26–111)	-0.20	**0.005**
ALAT, U/L (IQR)	32 (18—61)	-0.03	0.734
γ-GT, U/L (IQR)	59 (33—121)	-0.14	**0.049**

Continuous data are presented as median (interquartile range), and their association with cholinesterase was assessed by using Spearman–Rho correlation coefficient. ECMO, extracorporeal membrane oxygenation; IABP, intraaortic balloon pump; GFR, glomerular filtration rate estimated by using Cockcroft-Gault formula; SAPS II, simplified acute physiology score II.

Bold typefaces indicates statistical significance (*P* < 0.05).

Levels of butyrylcholinesterase showed a significant correlation with albumin, hypertension, hypercholesterolemia, and weak but significant inverse associations with EuroSCORE, ECMO duration, aspartate aminotransferase (ASAT), and γ-glutamyltransferase (Table 1). During a median follow-up time of 51 months (IQR, 34 to 71 months) corresponding to 4,197 overall months of follow-up, 65% of patients (*n* = 125) died. Of these, 89% of deaths had cardiovascular causes.

### Survival analysis

We identified a significant inverse association between higher butyrylcholinesterase levels and all-cause mortality with a crude hazard ratio (HR) of 0.50 (95% CI, 0.32 to 0.78; *P* = 0.002) as well as cardiovascular mortality with a crude HR of 0.44 (95% C,I 0.27 to 0.71; *P* = 0.001) when comparing the third with the first tertile. This effect was even more pronounced after adjustment for potential confounders with an adjusted HR of 0.44 (95% CI, 0.25 to 0.78; *P* = 0.005) for all-cause mortality and an adjusted HR of 0.38 (95% CI, 0.21 to 0.70; *P* = 0.002) for cardiovascular mortality. The results of the Cox regression analysis remained virtually unchanged after adjustment for other established risk factors (that is, previous coronary artery disease, chronic obstructive pulmonary disease, ECMO implantation, under cardiac massage, and ECMO-related complications (data not shown) and therefore not included in the final multivariate model.

**Figure 1:**
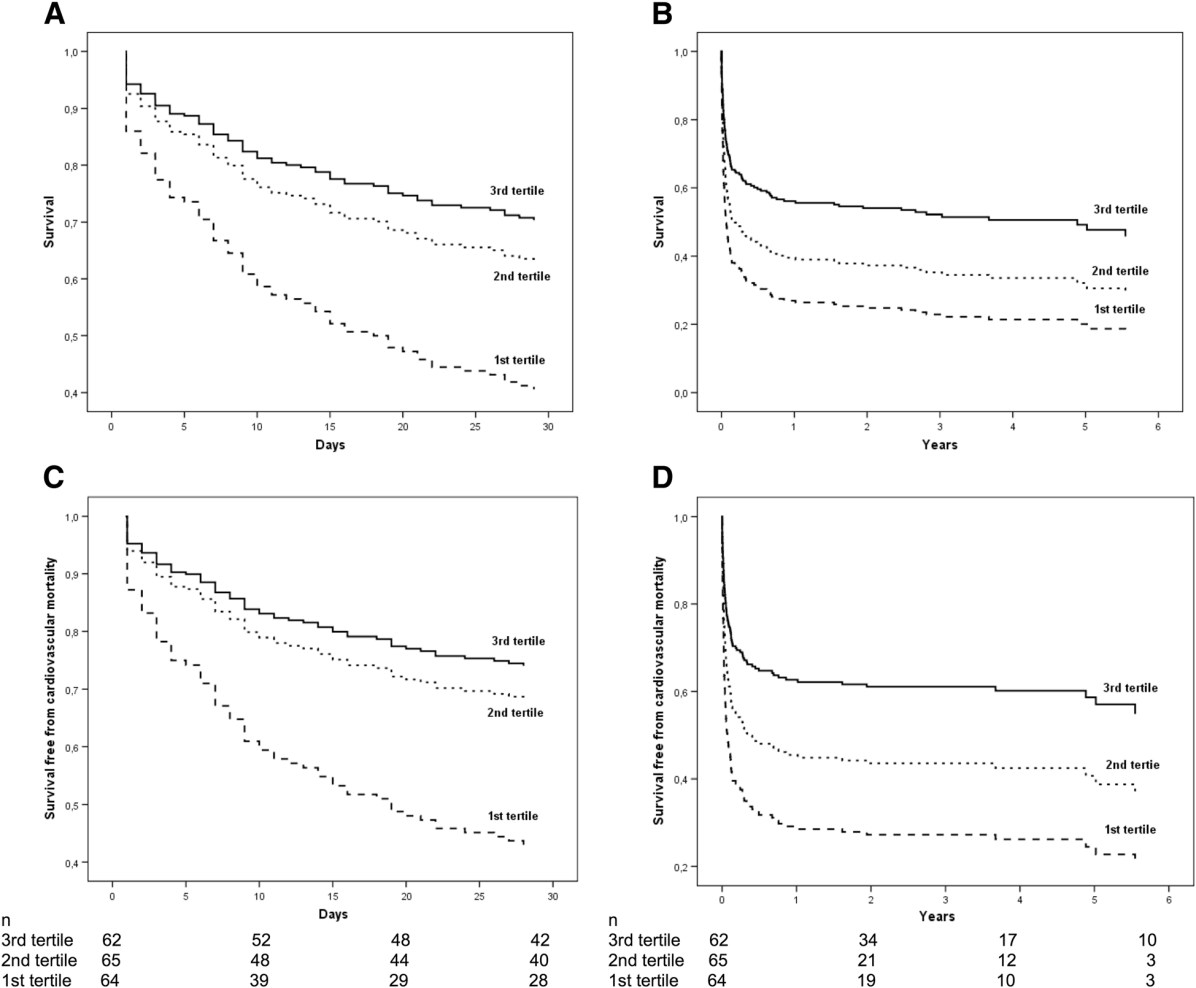
**Confounder-adjusted survival curves of all-cause and cardiovascular mortality according to tertiles of butyrylcholinesterase.** Adjusted survival curves demonstrated a significant decrease of 30-day (**A**, *P* = 0.017) and long-term (**B**, *P* = 0.019) all-cause mortality, as well as a significant decrease of 30-day (**C**, *P* = 0.008) and long-term (**D**, *P* = 0.007) cardiovascular mortality with increasing serum levels of butyrylcholinesterase.

Adjusted survival curves demonstrated a significant decrease of 30-day (*P* = 0.017; Figure 1A) and long-term (*P* = 0.019; Figure 1B) all-cause mortality, as well as a significant decrease of 30-day (*P* = 0.008; Figure 1C) and long-term (*P* = 0.007; Figure 1D) cardiovascular mortality with increasing serum levels of butyrylcholinesterase. Survival was increased in patients within the third tertile of butyrylcholinesterase compared with patients within the first tertile after 30 days (68% versus 44%), after 1 year (55% versus 30%), and after 6 years (47% versus 21%). A similar trend was observed for cardiovascular mortality.

## Discussion

The current study demonstrates for the first time a strong inverse association between serum butyrylcholinesterase levels and long-term mortality as well as short-term mortality in patients undergoing veno-arterial ECMO support following cardiovascular surgery. These associations were even more pronounced after adjustment for potential confounders.

Assessment of butyrylcholinesterase is an inexpensive, easily available laboratory marker that is routinely measured in many centers at hospital admission. Our data indicate that preoperative determination of serum butyrylcholinesterase provides essential information regarding the clinical outcome of patients requiring ECMO support. In regard of the high initial mortality of patients undergoing ECMO support following cardiovascular surgery [2,14-17], novel risk markers are critical for a more accurate risk assessment prior to surgery. Particularly, the strong predictive value for 30-day mortality as well as long-term mortality indicates the importance of serum butyrylcholinesterase for risk stratification. Considering the Kaplan Meier plots (Figure 1), the protective value of serum butyrylcholinesterase does not simply persist after 30 days but even increases over the observation period suggesting a sustained effect of butyrylcholinesterase on clinical outcome. Butyrylcholinesterase activity might be regarded as surrogate parameter for the patients’ general clinical conditions [18,19]. In elderly patients undergoing elective total hip replacement low preoperative serum butyrylcholinesterase activity has been identified to predict poor postoperative outcome [20]. The authors identified an inverse correlation of butyrylcholinesterase with total bilirubin and international normalized ratio, which is in line with prior studies suggesting low butyrylcholinesterase activity as surrogate marker for impaired liver synthesis and coagulation [3,21,22]. This association might be of major clinical relevance, considering that bleeding events are not only the most frequent ECMO-related complications but also the one with the greatest impact on mortality [23]. Patients requiring ECMO support represent a highly fragile population that is exposed to a strong ECMO-related systemic inflammatory response [24]. It is tempting to speculate that ECMO patients with decreased serum butyrylcholinesterase levels might have an increased vulnerability to stressors associated with ECMO support, due to reduced physiological reserves. However, as the exact function of serum butyrylcholinesterase is mainly unknown, the underlying pathophysiological mechanism has to be elucidated by future studies.

The enzyme butyrylcholinesterase is synthesized and secreted into blood by the liver and has been found to be positively associated with cardiovascular disease risk factors, including serum lipids, obesity, and hypertension [3,4]. In accordance with these data, we confirmed a significant correlation between serum butyrylcholinesterase levels and history of hypertension. Furthermore, we verified a close correlation of serum butyrylcholinesterase with albumin, which might be explained by the binding of butyrylcholinesterase to albumin [25]. However, after adjustment for hypertension and albumin the association of butyrylcholinesterase with mortality was even more distinct.

A potential limitation of our study is that our data reflect the experience of a single center. Furthermore, although our number of long-term survivors and median follow-up evaluated is among the highest reported to date, it might still not be sufficient to draw definitive conclusions.

We identified serum butyrylcholinesterase as a strong and independent inverse predictor of all-cause mortality and cardiovascular mortality in patients undergoing veno-arterial ECMO therapy following cardiovascular surgery. Preoperative risk factors such as advanced age, diabetes, adiposity, renal insufficiency, severe metabolic acidosis, chronic obstructive pulmonary disease, previous cardiac surgery, poor left-ventricular ejection fraction, and systolic blood pressure <90 mmHg have been identified as independent predictors for outcome in patients requiring ECMO support following cardiovascular surgery [10,14,15,26,27]. The current study complements the knowledge on risk stratification in these patients and represents a valuable addition for a comprehensive decision-making prior to ECMO implantation. These results are particularly compelling, because measurement of such a long-known biomarker as butyrylcholinesterase is inexpensive and routinely available in most laboratories. However, the exact pathophysiological mechanism underlying these associations has to be further elucidated. Considering the poor outcome of these patients, [2] an accurate preoperative risk prediction might be essential to reduce the unregulated use of ECMO which is associated with a disproportionately increase of health care costs and resources consumption. Therefore, the development of a clinically applicable mortality prediction score, including all of these clinical determinants as well as serum butyrylcholinesterase, has to be addressed by future studies. So far, ECMO patients with low butyrylcholinesterase levels should be considered at least as high-risk patients that may benefit from an intensified treatment and close check-ups after hospital discharge.

## Conclusions

The present study identified serum butyrylcholinesterase as a strong and independent inverse predictor of all-cause and cardiovascular mortality in patients undergoing veno-arterial ECMO therapy following cardiovascular surgery. These findings advance the limited knowledge on risk stratification in patients undergoing ECMO support and represent a valuable addition for a comprehensive decision-making prior to ECMO implantation.

## Key messages

• High mortality in patients undergoing venoarterial ECMO support after cardiovascular surgery.

• Preoperative serum butyrylcholinesterase levels are a strong and independent inverse predictor of all-cause mortality and cardiovascular mortality in patients undergoing ECMO therapy after cardiovascular surgery.

• Preoperative determination of serum butyrylcholinesterase levels might provide a valuable addition for a comprehensive risk-stratification before ECMO implantation.

## Abbreviations

ASAT: Aspartate aminotransferase; CABG: coronary artery bypass graft; ECMO: extracorporeal membrane oxygenation; eGFR: estimated glomerular filtration rate; EuroSCORE: European System of Cardiac Operative Risk Evaluation; FiO2: fractional inspired oxygen; SAPS: Simplified Acute Physiology Score.

## Competing interests

The authors declare that they have no competing interests.

## Authors’ contributions

KD made substantial contributions to conception and design, acquisition of data, as well as analysis and interpretation of data; drafting the article; and final approval of the version to be published. MW made substantial contributions to conception and design as well as acquisition of data; drafting the article; and final approval of the version to be published. KR made substantial contributions to conception and design as well as interpretation of data; revising the article critically for important intellectual content; and final approval of the version to be published. GH made substantial contributions to conception and design; revising the article critically for important intellectual content; and final approval of the version to be published. IL made substantial contributions to analysis and interpretation of data; revising the article critically for important intellectual content; and final approval of the version to be published. GM made substantial contributions to interpretation of data; revising the article critically for important intellectual content; and final approval of the version to be published. HK made substantial contributions to conception and design analysis as well as analysis and interpretation of data; revising it critically for important intellectual content; and final approval of the version to be published. BS made substantial contributions to conception and design and interpretation of data; revising it critically for important intellectual content; and final approval of the version to be published. AN made substantial contributions to analysis and interpretation of data as well as acquisition of data; revising the article critically for important intellectual content; and final approval of the version to be published. GG had overall responsibility including substantial contributions to conception and design, and analysis and interpretation of data; revising the article critically for important intellectual content; and final approval of the version to be published. All authors read and approved the final manuscript.
